# AMP‐activated protein kinase β1 or β2 deletion enhances colon cancer cell growth and tumorigenesis: Effects of AMPKβ1 or β2 deletion in colon cancer

**DOI:** 10.3724/abbs.2022086

**Published:** 2022-07-12

**Authors:** Fuli Shi, Zhimin Tang, Shanshan Jiang, Zhijuan Xiong, Wansi Zhang, Yuanjun Li, Hui Lin, Zhijun Luo, Ying Ying

**Affiliations:** 1 Jiangxi Medical Center for Major Public Health Events the First Affiliated Hospital of Nanchang University Nanchang 330006 China; 2 Jiangxi Province Key Laboratory of Tumor Pathogenesis and Molecular Pathology and Department of Pathophysiology School of Basic Medical Sciences Nanchang University Nanchang 330006 China; 3 Department of Immunobiology College of Life Science and Technology Jinan University Guangzhou 510632 China; 4 Institute of Hematological Research Shaanxi Provincial People’s Hospital Xi’an 710000 China; 5 Departments of Gastroenterology the First Affiliated Hospital of Nanchang University Nanchang 330006 China

**Keywords:** AMPKβ1, AMPKβ2, Warburg effect, tumorigenesis, colon cancer

## Abstract

Abnormal metabolism is a major hallmark of cancer and has been validated as a therapeutic target. Adenine monophosphate‐activated protein kinase (AMPK), an αβγ heterotrimer, performs essential functions in cancer progression due to its central role in maintaining the homeostasis of cellular energy. While the contributions of AMPKα and AMPKγ subunits to cancer development have been established, specific roles of AMPKβ1 and AMPKβ2 isoforms in cancer development are poorly understood. Here, we show the functions of AMPKβ1 and AMPKβ2 in colon cancer. Specifically, deletion of *AMPKβ1* or *AMPKβ2* leads to increased cell proliferation, colony formation, migration, and tumorigenesis in HCT116 and HT29 colon cancer cells. Interestingly, the AMPKβ1 and AMPKβ2 isoforms have slightly different effects on regulating cancer metabolism, as colon cancer cells with *AMPKβ1* knockout showed decreased rates of glycolysis-related oxygen consumption, while *AMPKβ2* deletion led to enhanced rates of oxygen consumption due to oxidative phosphorylation. These results demonstrate that functional AMPKβ1 and AMPKβ2 inhibit growth and tumorigenesis in colon cancer cells, suggesting their potential as effective targets for colon cancer therapy.

## Introduction

Adenine monophosphate‐activated protein kinase (AMPK), a well-known cellular energy sensor, functions as an essential component in the maintenance of energy homeostasis in normal cells and has been established as a major contributor (and hence therapeutic target) to both metabolic syndromes and cancers [1]. AMPK is a heterotrimeric complex containing a catalytic (α), a scaffold (β), and a regulatory subunit (γ) [2]. The N-terminal domain of the AMPKα subunit forms the catalytic core, while its C-terminal is responsible for binding and complex formation with the β and γ subunits [3]. Phosphorylation of Thr172 in the α subunit indicates AMPK activation and is required for the regulation of AMPK activity. An increasing body of evidence suggested that AMPK activation can also contribute to tumor cell metabolism, proliferation, survival, migration, and invasion, but most studies primarily focused on investigating the role of the AMPKα in promoting cancer cell growth and metastasis [ 4– 11] . Meanwhile, relatively few studies have examined the function of the AMPKβ subunit in tumorigenesis.

As the scaffold for AMPK, AMPKβ mediates α and γ subunit binding, serving as a “bridge” between α and γ in complex formation. The AMPKβ subunit has two isoforms, AMPKβ1 and AMPKβ2, encoded by the *PRKAB1* and *PRKAB2* genes, respectively, which can individually serve as scaffold subunits in different combinations with isoforms of the α and γ subunits. AMPKβ1 and AMPKβ2 are highly similar and share 71% protein sequence similarity. Recent studies have revealed distinct functions for AMPKβ1 and AMPKβ2 in addition to their role in the AMPK complex [ 12– 15] . In our previous study, we found that deletion of the *AMPKβ* subunit can promote the growth and migration of lung cancer cells [16]. However, it remains largely unknown if specific AMPKβ isoforms have different contributions to cancer development.

Colon cancer is a common malignancy originating from the digestive tracts and is the second leading cause of cancer-related death. The pathogenesis and mechanism of colon cancer are complex and related to several factors, including the interactions among genetic, environmental, and metabolism factors [17]. Several studies have demonstrated that reduced AMPKβ1 expression promotes the oncogenic ability of advanced ovarian cancer [ 18, 19] . High expressions of AMPKβ1 and AMPKβ2 are significantly related to increased 5-year survival among patients with non-Hodgkin lymphoma [20]. Additionally, overexpressed AMPKβ1 inhibits lung cell growth [21]. These findings indicate that AMPKβ1 and AMPKβ2 are related to cancer development. However, the role of AMPKβ1 and AMPKβ2 in colon cancer is unclear.

In the present study, we examined the differences in the potential roles of the AMPKβ1 and β2 isoforms in energy metabolism and colon cancer cell growth. By deleting *AMPKβ1* or *AMPKβ2* using the CRISPR/Cas9 genome editing, we investigated the individual effects of their deletion on tumor proliferation, migration, oxidative phosphorylation, and glycolysis in colon cancer cell lines. We also demonstrated the positive impacts of specific AMPKβ subunit deletion on tumor growth in nude mice *in* *vivo*.

## Materials and Methods

### Materials and reagents

The antibodies against β-actin, total AMPKα, and AMPKβ1 were purchased from Cell Signaling Technology (Danvers, USA), and anti-AMPKβ2 antibody was from Abcam (Cambridge, UK). Lipofectamine 3000 reagents were from Thermo Scientific (Waltham, USA). Glycolysis and Cell Mito Stress Test kits were from Agilent (Santa Clara, USA). Gibson Assembly Cloning kit was purchased from New England Biolabs (Ipswich, USA) to construct the guide RNA plasmid of AMPKβ1 and AMPKβ2 subunits.

### Cell culture and transfection

The colon cancer cells HT29 and HCT116 were purchased from American Type Culture Collection (ATCC; Manassas, USA). All these cell lines were cultured in RPMI 1640 medium supplemented with10% FBS (Hyclone, Logan, USA), 100 IU/mL penicillin, and 100 μg/mL streptomycin at 37°C and 5% CO _2_. Cells were transfected using Lipofectamine 3000 according to the instruction of the manufacturer.

### The Cancer Genome Atlas (TCGA) data analysis

The RNA-seq data for COAD including 41 normal tissues and 480 tumor tissues were obtained from the TCGA database ( https://portal.gdc.cancer.gov). The mRNA expressions of AMPKβ1 and AMPKβ2 were extracted from the data, and the corresponding expression was visualized using GraphPad Prism (GraphPad Software, San Diego. USA).

### Construction of guide RNA plasmids of AMPKβ1 and AMPKβ2 subunits

Using oligonucleotides shown in Table 1, we reconstructed the guide RNA plasmids of AMPKβ1 and AMPKβ2 isoforms according to the protocol described previously [16]. The β1 or β2 guide RNA plasmids were co-transfected with Cas9-GFP into HCT116 and HT29 cells, respectively. Then the cells were cultured for 2 days and selected using puromycin (2 μg/mL) for 3 days. Western blot analysis was used to identify the clones in which *AMPKβ1* or *AMPKβ2* was deleted.

**Table:** **Table 1** The sequences of guide RNAs

Gene	Primer sequence
β1 subunit guide RNA-1	Forward primer: 5′-CACCT471GGCCTGGCAGCATGATC488-3′ starting from W61 Reverse primer: 5′-AAACGATCATGCTGCCAGGCCA-3′
β1 subunit guide RNA-2	Forward primer: 5′-CACCA598AGTTTACTCCAGTTGTTGA579-3′ staring from ~K96 Reverse primer: 5′-AAACTCAACAACTGGAGTAAACTT-3′
β2 subunit guide RNA-1	Forward primer: 5′-CACCA355GAGTTTGTATCATGGCAGC374-3′ starting from ~E57 Reverse primer: 5′-AAACGCTGCCATGATACAAACTCT-3′
β2 subunit guide RNA-2	Forward primer: 5′-CACC449CTTGCCTCCTTCAGACCAGC429-3′ starting from ~D81 Reverse primer: 5′-AAACGCTGGTCTGAAGGAGGCAAG-3′

### Western blot analysis

The procedure of western blot analysis was performed as described previously [22]. Firstly, total proteins were extracted using RIPA lysis buffer (Thermo Scientific) containing proteinase inhibitors, phosphatase inhibitors, and PMSF. Secondly, protein concentration was measured using BCA Protein Assay kit (Thermo Scientific), and the protein samples were prepared using 6×SDS protein loading sample buffer. Thirdly, equal amounts of proteins (20‒40 μg/well) were loaded onto 10% SDS-PAGE gel and separated by electrophoresis, and then transferred onto PVDF membranes (Millipore, Billerica, USA). Subsequently, the membranes were blocked with 5% skimmed milk and incubated with primary antibodies, including anti-β-actin, total AMPKα, AMPKβ1, and AMPKβ2 antibodies, at 4°C overnight, followed by incubation with indicated HRP-conjugated secondary antibodies for 2 h at room temperature. Finally, protein bands were detected by using ECL western blotting substrate kit (Thermo Scientific).

### Cell viability assay

The cell viability was measured by using the CCK-8 assay kit (Everbright®Inc, Suzhou, China). Briefly, cells were seeded in 96-well plates at 4000 cells/well, and cultured for 24, 48, and 72 h, respectively. At the end of each time point, CCK-8 reagent was added to each well and incubated for an additional 4 h. Subsequently, the optical density was measured at 450 nm with a microplate reader (SpectraMax Paradigm; Molecular Devices, San Jose, USA).

### Wound healing assay

HT29 and HCT116 cells were plated in 6-well plates at 5×10 ^5^ cells/well, and incubated for 24 h. The culture medium was removed and replaced by PBS, and a scratch was made using a fine and sterilized 10-μL tip in each group. The detached cells were removed by washing three times with PBS to make sure no cells in the wound area. Then cells in the plates were cultured in normal medium and images were taken immediately after the first scratch (0 h), and then at 24 h and 48 h. The area of the scratch was analyzed using ImageJ software (NIH, Bethesda, USA).

### Transwell assay

Cell migration assays were performed in 12-well transwell units. After incubation at 37°C for 24 h, the cells were fixed using cold ethanol, and then stained with 0.1% crystal violet. The cells that invaded to the lower surface of the membrane were photographed with a Leica Microscope (Wetzlar, Germany). The units were dried at room temperature and the number of invaded cells was counted using ImageJ software.

### Colony formation assay

HT29 and HCT116 cells were seeded into 6-well plates at 300 cells/well, and continuously cultured for 14 days. At the end, the culture media were removed and the colonies were washed twice with PBS. Then, the colonies were fixed with 4% paraformaldehyde and stained with 0.1% crystal violet, followed by rinsing twice with PBS. Finally, images were taken and the number of colonies were counted.

### Cell metabolism assay

The glucose oxidation and glycolysis rates in HT29 and HCT116 cells were measured with the Seahorse Bioscience XF Analyzer (Agilent, Santa Clara, USA) using the Seahorse XF Glycolytic Rate Assay kit and Mito Stress Test kit (Agilent) respectively following the manufacturer’s instructions.

### Animal model of tumor

The xenograft mice model was used to investigate the effect of *AMPKβ1* or *AMPKβ2* knockout on tumorigenesis. Five to six weeks old male BALB/c nude mice (Nanjing University Biomedicine Institute, Nanjing, China) were subdivided into 3 groups, wild-type, *AMPKβ1*-KO, and *AMPKβ2*-KO groups. Cells (5×10 ^6^/100 μL in PBS) were injected subcutaneously into the back of nude mice. The tumor volumes were measured using calipers every other day from the eighth day after inoculation and calculated as follows: V=L×W ^2^/2, where W is the shorter diameter and L is the longer diameter. The animal experiments were approved by the Animal Use and Care Committee of Nanchang University (NCDXSYDWFL-2015097).

### Statistical analysis

Data and statistical analyses were performed using GraphPad Prism (v.8.0). The results were presented as the mean±standard deviation (SD). Two-tailed Student’s *t*-test was used to analyze the differences between two groups, and Two‐Way ANOVA was applied to determine the differences among different groups that had been split on two independent variables, such as volume and time. A *P* value less than 0.05 was considered statistically significant.

## Results

### Ablation of AMPKβ1 or AMPKβ2 leads to cell proliferation and colony formation

**Figure 1:**
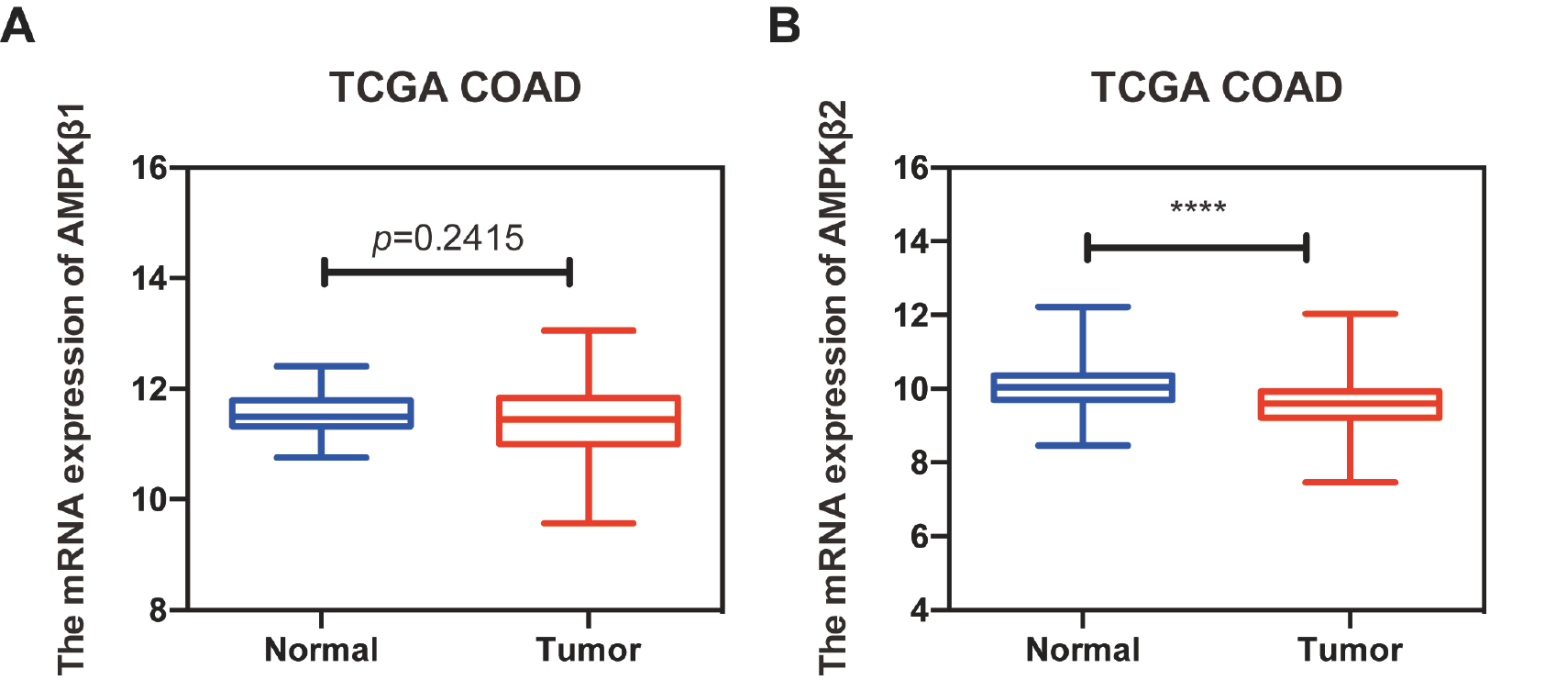
Figure 1 The mRNA expressions of *AMPKβ1* or *AMPKβ2* in colon cancer from TCGA (A) The expression of AMPKβ1. (B) The expression of AMPKβ2. Statistical analysis was performed using Student’s t-test. **** P<0.0001.

In order to investigate the potential biological functions of AMPKβ1 and AMPKβ2 in cancer development and progression, we analyzed the expressions of AMPKβ1 and AMPKβ2 in colon cancer by using TCGA data. Compared to that in the normal tissue, the mRNA expression of *AMPKβ2* in colon cancer tissues was significantly decreased ( *P*<0.0001; Figure 1). However, the AMPKβ1 expression was slightly decreased in colon cancer tissues, but there was no statistical significance between normal and colon cancer tissues ( Figure 1).

**Figure 2:**
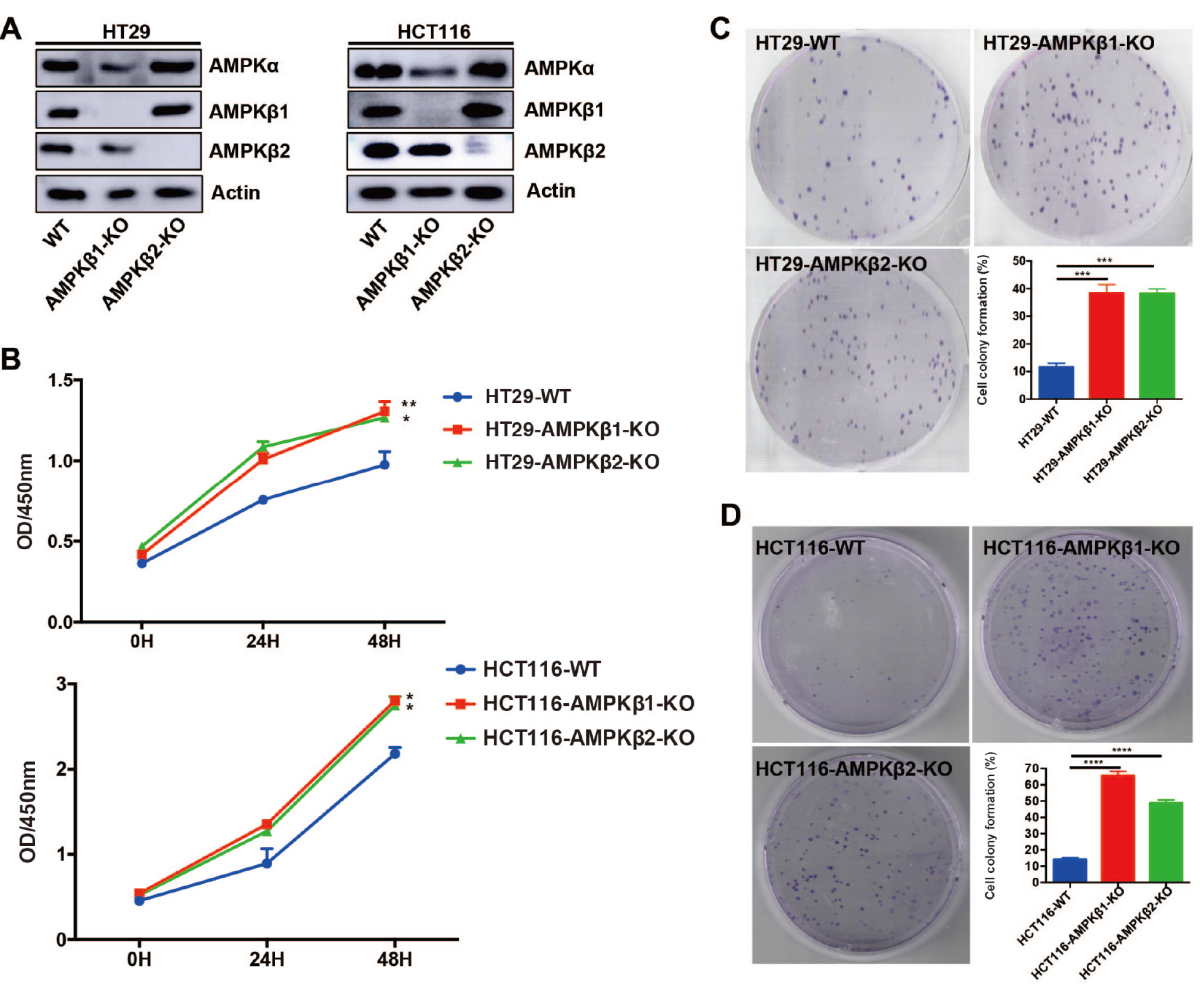
Figure 2 Ablation of *AMPKβ1* or *AMPKβ2* leads to cell proliferation and colony formation The HT29 and HCT116 cells were co-transfected with Cas9 and guide-RNAs targeting the AMPKβ1 or AMPKβ2. The AMPKβ1 or AMPKβ2 deletion cells were selected using puromycin. (A) Western blot analysis was used to confirm the expressions of AMPKβ1and AMPKβ2 in HT29 and HCT116 cells. (B) Cell viability was performed by CCK-8 assay. Cells were seeded in 96-well plates (4000 cells/well) and analyzed at 24, 48 and 72 h. Two‐way ANOVA was used for statistical analysis. * P<0.05, ** P<0.01. (C,D) Colony formation assays. Cells were seeded in 6 well-plates and cultured for 14 days. The colonies were stained and then images were captured. Student’s t-test was used to examine statistical significance. *** P<0.001, **** P<0.0001.

Furthermore, we used CRISPR/Cas9 to generate four cell lines carrying individual deletions for either *AMPKβ1* or *AMPKβ2*. Specifically, the HT29 and HCT116 cells were co-transfected with Cas9 and AMPKβ1 or AMPKβ2 guide-RNAs. Knockout (KO) of *AMPKβ1* or *AMPKβ2* was confirmed at the protein level by western blot analysis, revealing that AMPKα accumulation was significantly reduced in the *AMPKβ1*-KO cells, whereas *AMPKβ2* KO had no obvious effect on AMPKα protein expression level ( Figure 2A).

To determine whether AMPKβ1 or AMPKβ2 could indeed contribute to colon cancer development, CCK-8 and colony formation assays were used to examine the effects of their deletion on cell proliferation and colony formation in HT29 and HCT116 cells. The results of CCK-8 assay indicated that both *AMPKβ1*-KO and *AMPKβ2*-KO cells of the HCT116 and HT29 cell lines exhibited significantly higher proliferation rates than the wild-type (WT) cells of either respective cell line ( Figure 2B). Colony formation assays showed that the average colony diameter and number of colonies were both significantly greater for the *AMPKβ1*-KO and *AMPKβ2*-KO cells than those of their respective WT controls ( Figure 2C). These results suggested that AMPKβ1 and AMPKβ2 both appear to negatively impact the proliferation and colony formation of colon cancer cells.

### *AMPKβ1* or *AMPKβ2* deletion leads to enhanced migration of colon cancer cells

**Figure 3:**
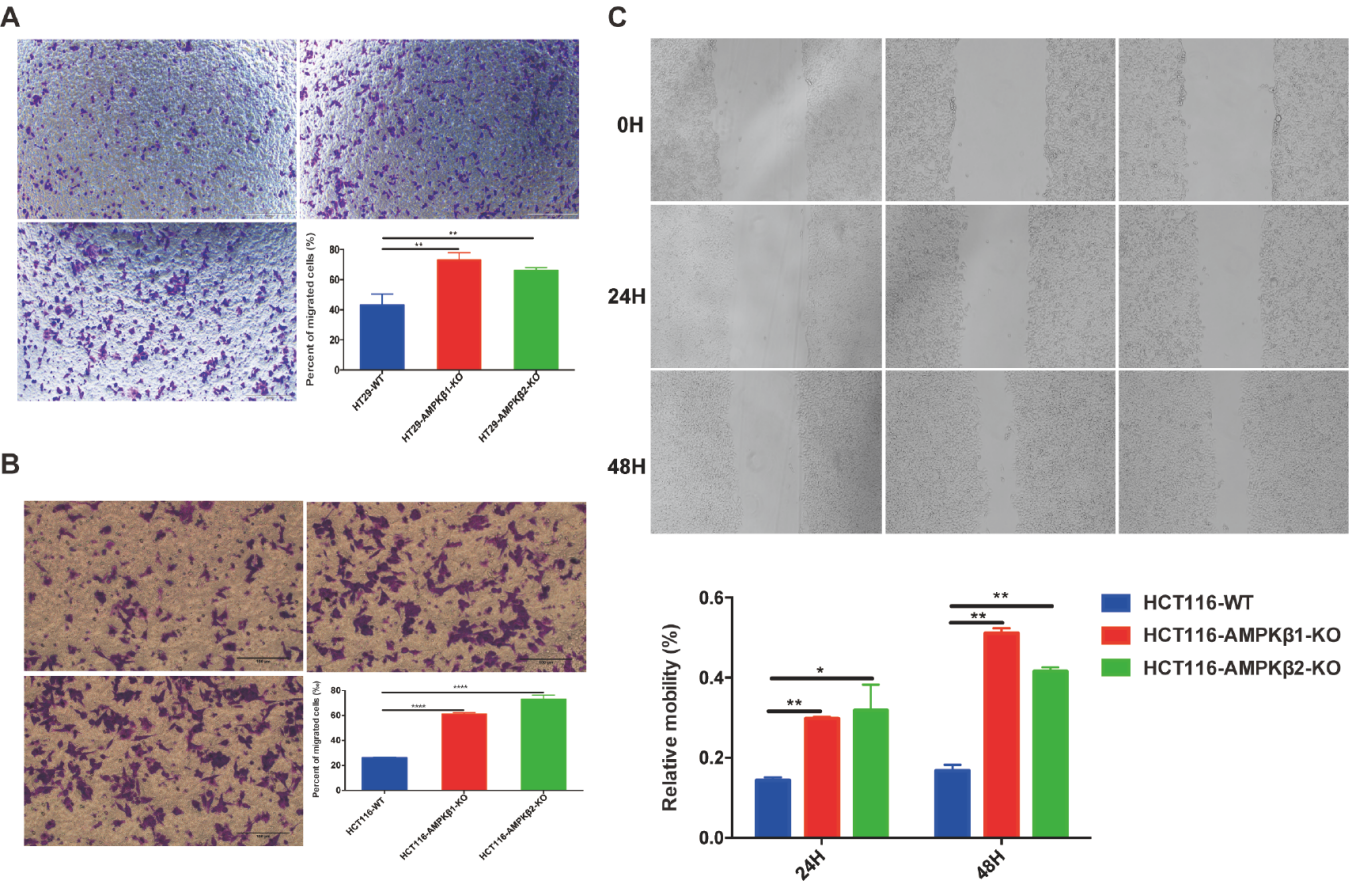
Figure 3 *AMPKβ1* or *AMPKβ2* deficiency accelerates cell migration of HCT116 and HT29 cell lines Transwell assays showed that AMPKβ1 or AMPKβ2 subunit deletion promoted HT29 (A) and HCT116 (B) migration ability. Deletion of AMPKβ1 or AMPKβ2 subunit promoted HCT116 (C) cell migrating into the scratching area tested by wound healing assay. * P<0.05, ** P<0.01, **** P<0.0001.

Given that metastasis is a critical step in cancer progression, we next sought to determine if the knockout of either *AMPKβ1* or *AMPKβ2* isoform positively impacts their motility and invasion by transwell and wound healing assays. The transwell assay showed that *AMPKβ1*-KO or *AMPKβ2*-KO cells exhibited markedly higher levels of migration than WT HT29 cells ( Figure 3A) or WT HCT116 cells ( Figure 3B). Similarly, wound healing assays confirmed that *AMPKβ1* or *AMPKβ2* deletion resulted in significantly increased migration compared to WT HCT116 cells ( Figure 3C). Notably, both WT and KO HT29 cells showed poor adherence, thus preventing their use in wound healing assays. Collectively, these data indicated that KO of either *AMPKβ1* or *AMPKβ2* subunit resulted in enhanced migration of colon cancer cells.

### *AMPKβ1* or *AMPKβ2* depletion promotes tumor growth in nude mice

**Figure 4:**
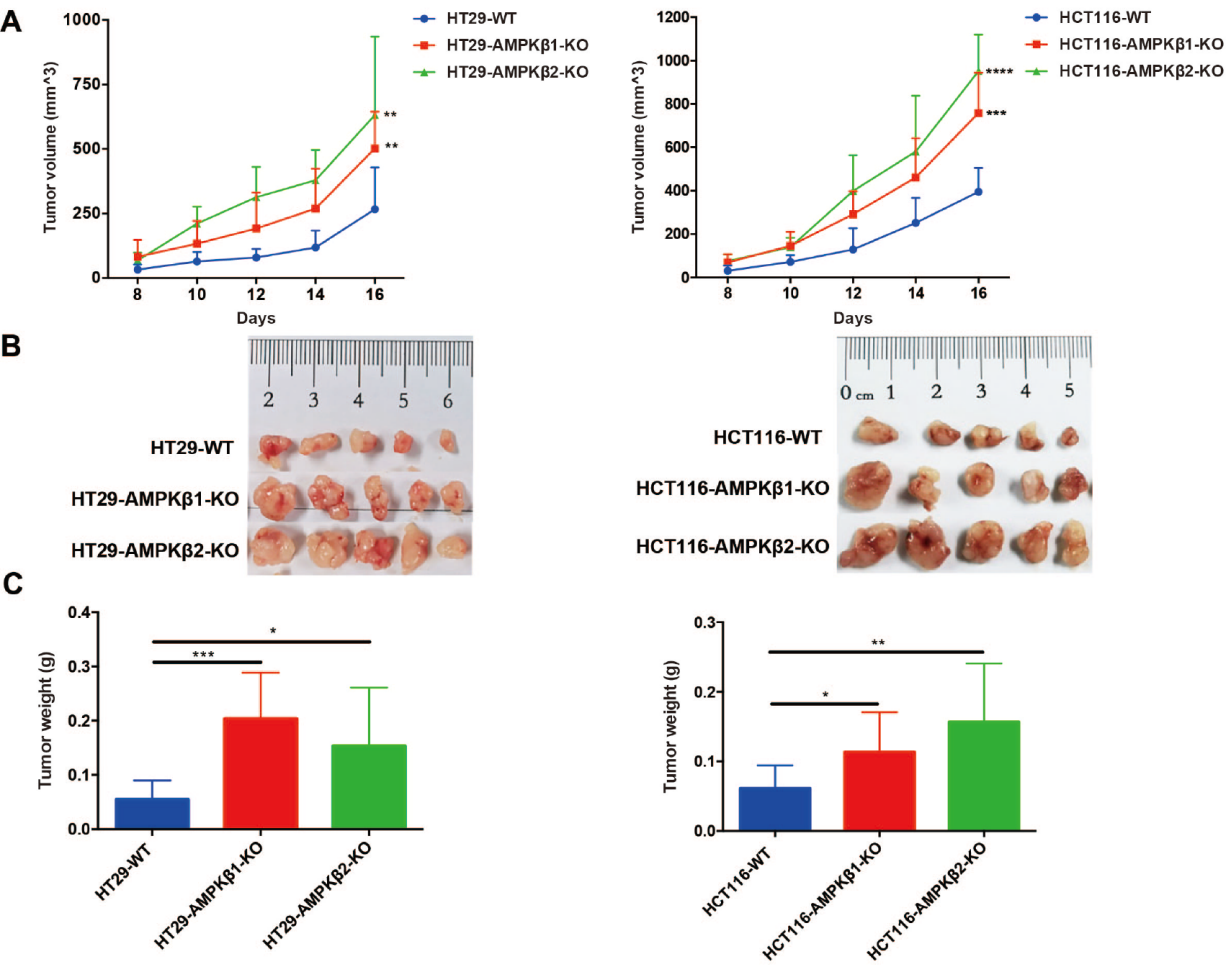
Figure 4 *AMPKβ1* or *AMPKβ2* depletion promotes tumor growth in nude mice AMPKβ1 or AMPKβ2 depletion or wild-type HCT116 and HT29 cell lines were injected subcutaneously into the back of nude mice. (A) Tumor growth was monitored every other day. The statistical analysis of comparative tumor growth between each group was analyzed by using two‐way ANOVA. (B) The representative image of the tumor. (C) The weight of tumors. Statistical analysis of the weight of the tumors was tested by Student’s t-test. * P<0.05, ** P<0.01, *** P<0.001, **** P<0.0001.

To evaluate the effects of *AMPKβ1*-KO and *AMPKβ2*-KO *in vivo*, we subcutaneously implanted AMPKβ1-KO, AMPKβ2-KO, WT HCT116, or WT HT29 cells (5×10 ^6^ cells) in nude mice. Tumor sizes were then measured with a caliper every other day following implantation, and the mice were sacrificed and photographed on day 16 ( Figure 4A). As shown in Figure 4B, the tumors originated from *AMPKβ1*-KO or *AMPKβ2*-KO cells grew faster than tumors derived from either WT colon cancer cell line. The average tumor volume and weight were also significantly higher in mice bearing *AMPKβ*1-KO or *AMPKβ*2-KO tumors compared to those of WT tumor-bearing mice ( Figure 4C). These results demonstrated that the tumor-promoting effects of *AMPKβ1*-KO or *AMPKβ2*-KO could be recapitulated *in vivo*.

### *AMPKβ1* or *AMPKβ2* deletion results in metabolic dysregulation

**Figure 5:**
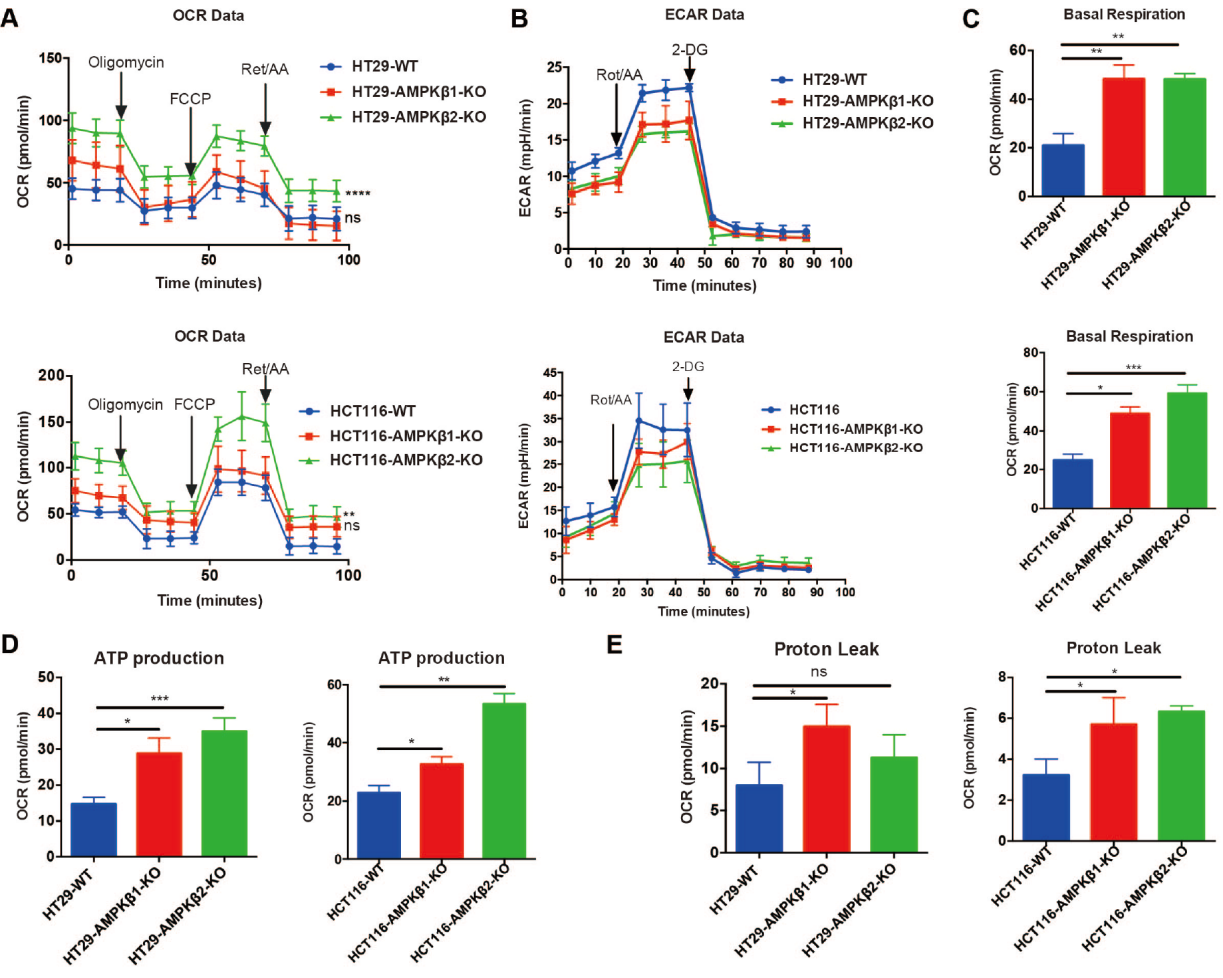
Figure 5 Effects of *AMPKβ1* or *AMPKβ2* on glycolysis and oxidative phosphorylation Changes in cellular metabolisms of AMPKβ1 or AMPKβ2 depletion or wild-type HCT116 and HT29 cell lines were assessed using Seahorse Bioscience XF. (A) The Oxygen consumption rate (OCR) of cells. (B) The extracellular acidification rate (ECAR) of cells. (C) OCR consumed by basal respiration in HT29 and HCT116 cells. (D) OCR consumed by ATP production in HT29 and HCT116 cells. (E) OCR consumed by proton leak in HT29 and HCT116 cells. Student’s t-test was used to interpret the statistical significance. * P<0.05, ** P<0.01, *** P<0.001.

Our previous study demonstrated that AMPK can inhibit cancer cell growth and tumorigenesis by regulating mitochondrial metabolic homeostasis [16]. To assess if *AMPKβ1* and *AMPKβ2* deletion promote tumorigenesis via metabolic regulation, we detected glucose metabolism in colon cancer cells harboring AMPKβ1 or AMPKβ2 KO using an XF Seahorse analyzer to measure the rate of oxygen consumption (OCR) and extracellular acidification rate (ECAR). OCR reflects the capacity of oxidative phosphorylation, and ECAR can be used to quantify the cellular level of glycolysis. Our results showed that deletion of *AMPKβ1* had no effect on the OCR, but *AMPKβ2* deletion significantly increased OCR in both HCT116 and HT29 cells compared to those of WT cells ( Figure 5A). To our surprise, ECAR values were decreased in both *AMPKβ1*-KO and *AMPKβ2*-KO cancer cells compared to those in WT cells ( Figure 5B).

To explore other potential mechanistic effects of *AMPKβ*1 and *AMPKβ*2 on cancer metabolism, we exploited the relationships among OCR and basic respiration, ATP production, and proton leakage. To this end, we estimated the OCR for each of these metabolic functions in WT and KO cancer cells using the following formula: Basal respiration = ATP-linked respiration + proton leak-linked respiration. The basal respiration-related OCR ( Figure 5C) and the ATP production-related OCR ( Figure 5D) were both significantly increased in *AMPKβ1*-KO and *AMPKβ2*-KO cells compared to those in WT cells. Notably, we found that proton leakage-related OCR ( Figure 5E) was significantly higher in WT cells than in *AMPKβ1*-KO and *AMPKβ2*-KO HCT116 cells, while only *AMPKβ1*-KO but not *AMPKβ2*-KO HT29 cells had higher predicted OCR due to proton leakage. These data suggest that the effects of loss of AMPKβ1 or β2 function on cancer cell progression are mediated by metabolic dysregulation.

## Discussion

AMPKβ1 and AMPKβ2 isoforms are essential structural subunits required for AMPK activity in mitochondrial metabolism, although the differences in the physiological and pathological roles between the AMPKβ1 and AMPKβ2 isoforms remain unclear, especially in colon cancer development. For example, *AMPKβ1* deletion is linked to reduced survival and enhanced oncogenic capacity in different cancers [ 12, 18, 19] , although it is unknown whether AMPKβ2 can also participate in tumorigenesis. This study provides evidence supporting major roles for both AMPKβ1 and AMPKβ2 in the pathogenesis and progression of colon cancer. We observed that the expression of AMPKβ2 was decreased in the colon cancer tissues, and deletion of either *AMPKβ1* or *AMPKβ2* resulted in elevated cancer cell proliferation, colony formation, tumor cell migration, and *in vivo* tumorigenesis in two distinct colon cancer cell lines. Our findings are in agreement with our preliminary data showing that lung cancer cells harboring deletions for either subunit exhibit similar growth and proliferation phenotypes (data not shown). These findings strongly suggest that both *AMPKβ1* and *AMPKβ2* isoforms can inhibit tumorigenesis in cancer cells.

Intriguingly, we found that some aspects of AMPKβ1 function differ from those of AMPKβ2. For example, *AMPKβ1* deletion results in decreased protein level of AMPKα, whereas deletion of the *AMPKβ2* isoform produces no obvious effects on the expressions of other AMPK subunits. In our previous study, we found that simultaneous ablation of both the AMPKβ1 and AMPKβ2 subunits reduced the expression of α subunit, although we could not determine which isoform deletion caused the reduction of AMPKα. Here, we provide evidence that the AMPKβ1 isoform apparently participates in controlling the accumulation of AMPKα, a finding consistent with another previous investigation [23]. Additionally, we demonstrated that loss of the AMPKβ2 subunit leads to an increase in compensation for the AMPKβ1 subunit, which is also in agreement with findings of previous studies [ 24, 25] . It is therefore reasonable to conclude that AMPKβ1 plays a more important role than AMPKβ2 in maintaining an intact AMPK complex. However, the mechanism of AMPKβ1 action in regulating AMPKα expression remains unclear and requires further investigation.

Metabolic dysregulation is a hallmark of cancers. Warburg and colleagues showed that cancer cells preferentially utilize glycolysis rather than oxidative phosphorylation for energy production [26]. Targeting tumor glycolysis has thus become a relatively common therapeutic strategy for cancer treatment. Consistent with our previous report [16], we observed that knockout of *AMPKβ1* decreased the glycolysis capacity of cancer cells, but caused no significant change in oxidative phosphorylation in cancer cells. One explanation is that lack of AMPKβ1 causes reduced AMPK activity, which modulates extra-mitochondrial glucose catabolism [16]. Surprisingly, loss of AMPKβ2 not only reduces glycolytic activity but also promotes oxidative phosphorylation in cancer cells. Notably, our **in viv*o* experiments in nude mice showed that tumors derived from cells harboring AMPKβ2 deletion grew faster than those derived from cells with AMPKβ1-KO. Although these results seem controversial due to β2 enhancement of cellular respiration, other recent studies have shown that tumor cells with enhanced oxidative capacity can rapidly proliferate, and that some small-molecule inhibitors of oxidative phosphorylation also inhibit tumor growth [ 27– 32] .

Although the AMPKβ1 and AMPKβ2 isoforms have highly similar protein sequences, their different functions in metabolic regulation have been reported in several studies. For instance, suppression of AMPKβ2 expression with siRNA inhibits lipid accumulation, secretion, and cellular levels of adiponectin during 3T3-L1 adipogenesis, but silencing of *AMPKβ1* does not show such effects, thus indicating that AMPKβ2 contributes to adipogenesis [15]. Moreover, Milbrandt *et al*. [24]found that *AMPKβ2*-deficient animals are at high risk of high fat diet-induced metabolic syndromes including hyperglycemia, glucose intolerance, and insulin resistance. However, animals with loss of *AMPKβ1* exhibit reduced food intake and body mass, improved insulin sensitivity, as well as protection against diet-induced obesity and hepatic steatosis [23]. Our current findings unveiled differences in the effects of *AMPKβ1* or *AMPKβ2* deletion on metabolic dysregulation in cancer cells: deletion of *AMPKβ2* not only decreases glycolysis but also enhances oxidative phosphorylation in cancer cells, while *AMPKβ1* deletion only results in reduced glycolysis. However, the mechanism underlying these functional differences in their effects on cancer metabolism remains unclear.

In conclusion, our study shows that the deletion of either *AMPKβ1* or *AMPKβ2* potentiates tumorigenesis and enhances cancer cell proliferative and pro-metastatic functions. The results thus indicate that therapeutic targeting of AMPKβ1 and AMPKβ2 may be effective strategies for limiting tumor cell proliferation.
